# Prevalence and Correlates of Poor Sleep Quality Among Psychiatry Physicians in Saudi Arabia: A Cross‐Sectional Study

**DOI:** 10.1002/hsr2.70170

**Published:** 2024-10-30

**Authors:** Najim Z. Alshahrani, Abdullah M. Alarifi, Wejdan Saqer Alotaibi, Afnan Abdulrahman Alsayed, Khalid Sultan Latif Alwasm, Alaa Abdulkarim Alhunti, Lana Alaa AlDahleh, Meaad Mohammed A Alshahrani, Abdalrhman M. Albeshry, Mohammed A. Aljunaid

**Affiliations:** ^1^ Department of Family and Community Medicine, Faculty of Medicine University of Jeddah Jeddah Saudi Arabia; ^2^ Deputyship of Public Health Ministry of Health Riyadh Saudi Arabia; ^3^ Psychiatry Department Prince Mohammed bin Abdulaziz Hospital Riyadh Saudi Arabia; ^4^ Psychiatry Department Forces Security Hospital Riyadh Saudi Arabia; ^5^ Psychiatric Physician Prince Mutaib bin Abdulaziz Hospital Sakaka Al Jouf Saudi Arabia; ^6^ Psychiatry Department, Alamal Hospital Qassim Health Cluster Buraidah Saudi Arabia; ^7^ Psychiatry Physician Joint Psychiatry Residency Training Program, Eradah Riyadh Saudi Arabia; ^8^ Mental Health Department King Fahad Medical City Riyadh Saudi Arabia

**Keywords:** depression, factors, psychiatry physicians, Saudi Arabia, sleep quality

## Abstract

**Background and Aims:**

Sleep issues pose a significant burden to public health and well‐being in Saudi Arabia. However, research evidence on sleep health among psychiatry physicians in this territory is limited. Therefore, to bridge the research gap, this study was designed to assess the prevalence and predictors of poor sleep quality among psychiatry physicians in the country.

**Methods:**

This cross‐sectional study included 554 psychiatry physicians in Saudi Arabia from March to August 2023. Data were collected via online through a structured questionnaire (Google survey form). Sleep quality, the outcome variable of our study, was evaluated with the Pittsburgh Sleep Quality Index (PSQI; 19 items). Independent variables included sociodemographic and behavioral characteristics, sleep habits, major depression (assessed with Patient Health Questionnaire‐9), and anxiety (measured with Generalized Anxiety Disorder‐7) symptoms. Binary logistic regression analysis was performed to identify the correlates of poor sleep quality.

**Results:**

Based on the PSQI, 61.3% of the study participants had poor‐quality sleep (age range: 24–56 years, male: 48.0%). The adjusted model revealed that male participants (AOR = 2.80, 95% CI = 1.70–4.61) and those who had on‐call duties ≥ 2 times per week (for three/four per week: AOR = 3.41, 95% CI = 1.89–6.14) were at higher risk of developing poor sleep quality compared to their respective counterparts. Participants with depressive symptoms (AOR = 3.46, 95% CI = 1.60–7.48) and smoking habits (AOR = 3.47, 95% CI = 1.32–9.08) had higher odds of developing poor sleep quality than their counterparts. Moreover, participants who always used their smartphone/laptop before going to bed were more likely to have poor sleep quality than those who never used such (AOR = 3.15, 95% CI = 1.31–7.60).

**Conclusion:**

Poor sleep quality is extremely prominent among psychiatry physicians in Saudi Arabia. Male sex, higher on‐call duty, smoking habits, depression, and smartphone/laptop use before bedtime were significantly associated with poor sleep quality. These findings emphasize the need for sleep‐health promotion interventions for Saudi psychiatry physicians.

## 1 Background

Sleep is required for the proper functioning of numerous psychological and physiological mechanisms in our body [1]. Adequate sleep is vital to optimal human health and is becoming recognized as a key parameter for cognitive, emotional, and physical health [2, 3]. On the contrary, poor sleep quality and sleep disorders harm both physical and psychological wellness and the immune system [1]. Sleep disturbance and deprivation are linked to negative health outcomes such as heart disease, weight gain, psychiatric issues, and neurological disorders [4, 5, 6].

Sleep quality is an extensive phenomenon that is not easy to assess typically [7]. Consequently, the empirical reliability and validity of existing and upcoming investigations are entirely reliant on the methodological approach employed to measure the quality of sleep. There are different subjective (e.g., Consensus Sleep Diary and Pittsburgh Sleep Quality Index [PSQI]) and objective measures (e.g., polysomnography and actigraphy) to assess sleep quality [7]. Of all the measures, PSQI‐19 is a widely used and validated tool for screening sleep quality that can classify individuals as “good” or “poor” sleepers [8].

Healthcare professionals are one of the vulnerable groups to experience substandard sleep because of their professional duties to the people and country (such as providing treatment to care‐seeking people, routine work, emergency shifts, etc.). Earlier systematic review studies, for example, have shown that healthcare workers had poorer‐quality sleep than the general public amid COVID‐19 [9, 10]. Healthcare practitioners' sleep patterns have been studied in different countries [11, 12, 13]. A study conducted among resident physicians (*n* = 514) in Damascus (the capital of Syria) reported that more than three‐quarters (79.5%) were poor sleepers based on the PSQI [11]. A recent study (*n* = 150) investigated the sleep quality of Egyptian medical residents and reported a higher prevalence of poor sleepers (poor sleepers: 96.7% according to the PSQI) [12].

Several factors have been explored to be connected with poor levels of sleep or sleep disorders among healthcare professionals. These include (i) social and health‐related factors: age, sex, marital situation, long‐term illness, past experience of insomnia, social assistance, and so forth [10, 13]; (ii) occupational factors: department of service, on‐call scheduling, night duty, professional experience, working hours, and so forth [10, 14]; and (iii) psychological factors: anxiety, depression, stress, and so forth [10, 14]. Moreover, sleep quality can be affected by environmental, behavioral, and lifestyle factors such as daily workout levels, active smoking, and passive smoking [15, 16].

Sleep issues have been rising drastically in Saudi Arabia, receiving considerable attention from the healthcare systems and policymakers [13, 17, 18]. Numerous studies have been conducted on sleep quality among different vulnerable groups like students [15, 19], medical/commission residents [13, 20], and diabetes patients [21] in Saudi Arabia. For instance, a Saudi‐based study undertaken among medical residents showed a greater proportion of poor‐quality sleep (86.3% and *n* = 1205) [13]. Another recent study reported a 70.4% prevalence of poor sleep quality among healthcare professionals (physicians and nurses, *n* = 395) in Saudi Arabia [22]. However, research evidence on sleep health among psychiatry physicians in the country is limited. Given that importance, our study aims to bridge the research gap by exploring the sleep quality among psychiatry physicians in Saudi Arabia. This research would provide baseline data for policymakers and public health practitioners. Thus, the study was designed with the following objectives: (i) to assess sleep quality (good vs. poor) among psychiatry physicians in Saudi Arabia, and (ii) to examine the predictors of poor sleep quality.

## 2 Materials and Methods

### 2.1 Study Design and Ethical Compliance

This was a cross‐sectional investigation. The survey was conducted from March 15 to August 15, 2023. All study procedures involving human participants followed the Declaration of Helsinki. In addition, ethical authorization was taken from the Research Ethics Committee of Security Forces Hospital Program in the holy Capital (approval number: ECM#0546‐131222). Informed consent (i.e., electronic signature) was taken from all participants. The consent document precisely addressed the study's purposes and implications. It was made clear that participation was completely voluntary and anonymous. Furthermore, participants were guaranteed that sensitive data would be kept private. No incentives were given to the participants for participating in the study.

### 2.2 Study Subject and Data Collection Procedure

We performed this survey among psychiatry physicians (Saudi nationality) in Saudi Arabia to evaluate sleep quality and its correlates. The minimum required samples (*n*) for this study were calculated using the single sample proportion test. The calculation was done by considering a 5% margin of error (*d* = 0.05), a 95% confidence interval (CI) (*Z* = 1.96), and an expected prevalence of poor sleep quality of 70.4% based on the previous literature (*p* = 0.704) [22].

Accordingly, the minimum sample size,

$$n=\frac{z^{2}\times p\times (1‐p)}{d^{2}}=\frac{{\left(1.96\right)}^{2}\times 0.704\times (1‐0.704)}{{\left(0.05\right)}^{2}}=320.21\approx 320.$$

We added more samples than the calculated minimum numbers to strengthen the study's validity and precision level. Thus, the final sample of this study comprised 354 psychiatry physicians.

We used a web‐based approach to gather data so that study participants could respond at their convenience, as they may be occupied with professional obligations. Data were collected online through a structured questionnaire, which was formed in a Google Doc file. The survey link was sent to the psychiatry physicians via email. To reach the study participants, the research team used the following procedures: (i) the research team communicated with the authorities of the Saudi Commission for Health Specialties (SCFHS) and obtained psychiatry physicians' identifying information (such as names and email addresses), and (ii) then, the team sent the survey invitation via email to the participants. Instructions on how to agree and engage in this research were included in the body of the email. In the event of a delayed response, they were gently reminded with a weekly follow‐up email. If a participant did not respond, he or she was followed up for two consecutive weeks. Approximately 10–12 min were required to finish the survey.

### 2.3 Survey Contents and Measures

The survey tool consisted of five subparts: (i) sociodemographic and behavioral information, (ii) sleep‐related habits, (iii) assessment of major depression symptoms, (iv) evaluation of anxiety symptoms, and (v) assessment of sleep quality.

Participants' sociodemographic and behavioral information such as age, sex (male vs. female), marital status (single vs. married vs. widow, divorced, or separated), having children (yes vs. no), monthly income (< 15,000 Saudi Riyal [SAR] vs. 15,001–20,000 SAR vs. > 20,000 SAR), work classification (resident vs. specialist vs. consultant), institution of work (government vs. private), frequency of on‐call in the last month (one per week vs. two per week vs. three/four per week), current smoking status (yes vs. no) and self‐reported body mass index (underweight vs. normal weight vs. overweight/obese) were included. Participants' ages were recorded as a continuous measure and then split into four groups (24–29, 30–40, 41–50, and > 50 years). To enhance readability, we are clarifying the meanings of resident, specialist, and consultant. Residents are the most junior grade of hospital doctors who practice and are supervised by consultants and specialists and are still under training in a residency program for 4 years after their medical school graduation. Consultants are the most senior grade of hospital doctors and are responsible for leading a team. Specialists or specialty doctors (SAS Doctors) will have had at least 4 years of postgraduate training including at least 2 in their specialty.

In addition, information on sleep‐related habits with four possible options (always, usually, sometimes, or never) was obtained. These habits include using the sleeping room for entertainment/study, using a smartphone or computer before bedtime, exercising in the evening, overeating at night, and consuming caffeinated drinks at night [11].

Participants' major depression symptoms were assessed using the Patient Health Questionnaire (PHQ‐9), which consists of nine items with Likert‐scale responses ranging from not at all to nearly daily (0–3) [23]. The total score ranges from 0 to 27, with scores ≥ 10 indicating depressive symptoms [24]. A good level of internal consistency was found for this scale (Cronbach's *α* = 0.79). Previously, this scale was validated and used in several quantitative research in Saudi Arabia [25, 26, 27].

Individuals' anxiety symptoms were assessed using the validated Generalized Anxiety Disorder (GAD‐7) tool [27, 28]. The GAD‐7 scale is made up of seven items, which is rated on a four‐point Likert scale from 0 (never) to 3 (nearly every day) (total score: 0–21). In this investigation, participants who scored ≥ 10 were identified as having anxiety symptoms [29]. In our sample, this scale had a good level of reliability (Cronbach's *α* = 0.77).

Participants' sleep quality, the outcome variable of this study, was measured by a 19‐item PSQI [8]. This tool assesses the subjective sleep quality and patterns over the preceding month. This tool is subdivided into the following components: (i) subjective sleep quality, (ii) sleep latency, (iii) sleep duration, (iv) habitual sleep efficiency, (v) sleep disturbance, (vi) use of sleep medication, and (vii) daytime dysfunction. Each component could get a score between 0 and 3. An individual's seven component ratings, which vary from 0 to 21, are added up to obtain a global PSQI score for sleep quality. A score of > 5 on the global PSQI represented a criterion for poor sleep quality and a score ≤ 5 was marked as good sleep quality. Previous epidemiological investigations used the PSQI as a sleep quality screener among a variety of subpopulations worldwide [30, 31], including Saudi Arabia [13, 17, 18]. In the present data, Cronbach's *α* for this PSQI scale was 0.88 (i.e., excellent level of internal consistency).

### 2.4 Statistical Analysis

Enumerative parameters such as responses and percentages were computed to summarize the study variables. A chi‐square test was performed to observe the variation in the two categories of the dependent variable (sleep quality: good vs. poor) by the explanatory characteristics. A multivariate binary logistic regression model was fitted to identify the correlates of poor sleep quality. To identify sociodemographic, behavioral, and mental health‐related correlates of poor sleep quality, 12 independent variables were included in the adjusted model. Another adjusted regression model was constructed, including five variables related to participants' sleep habits, to examine how sleep‐related habits were associated with poor sleep quality. Key assumptions (such as multicollinearity and outliers) were checked before fitting the adjusted model. The fitness of the adjusted regression model was confirmed by the Hosmer and Lemeshow test. Odds ratios and 95% CIs were computed. The significance level was defined by *p* values less than 0.05 (two‐tailed). STATA (BE version 16.0; StataCorp) was used for all analyses.

## 3 Results

### 3.1 Sociodemographic, Behavioral, and Mental Health‐Related Characteristics

Of 354 participants, more than half of them were female (52.0%). The participants' ages ranged from 24 to 56 years old, with about half falling between 24 and 29 years (49.7%). The majority of the psychiatry physicians worked as residents (50.2%), and the rest of them were specialists (29.7%) or consultants (20.1%). Around one‐third of the participants (35.3%) had on‐call duty three or four times per week in the past month. Only 12.1% of them were smokers. Less than half of the participants (44.6%) had depressive symptoms based on the PHQ‐2 screener. Participants' sociodemographic, behavioral, and mental health‐related characteristics are demonstrated in Table 1.

**Table 1:** Sociodemographic, behavioral, and mental health‐related characteristics of study participants (*n* = 354).

Variables	Frequency	Percentage
Sex		
Male	170	48.0
Female	184	52.0
Age (in years)		
24–29	176	49.7
30–40	105	29.7
41–50	63	17.8
> 50	10	2.8
Marital status		
Single	171	48.3
Married	163	46.0
Widow, divorced, or separated	20	5.6
Having children		
Yes	140	39.5
No	214	60.5
Income (monthly)		
< 15,000 SAR	34	9.6
15,001–20,000 SAR	207	58.5
> 20,000 SAR	113	31.9
Work classification		
Resident	178	50.2
Specialist	105	29.7
Consultant	71	20.1
Institution of work		
Government	284	80.2
Private	70	19.8
Frequency of on‐call in the last month		
One per week	164	46.3
Two per week	65	18.4
Three or four per week	125	35.3
Current smoking status		
Yes	43	12.1
No	311	87.9
Self‐reported body mass index		
Underweight	55	15.5
Normal weight	167	47.2
Overweight/obese	132	37.3
Having depressive symptoms		
Yes	158	44.6
No	196	55.4
Having anxiety symptoms		
Yes	133	37.6
No	221	62.4

### 3.2 Prevalence and Patterns of Sleep Quality

**Figure 1:**
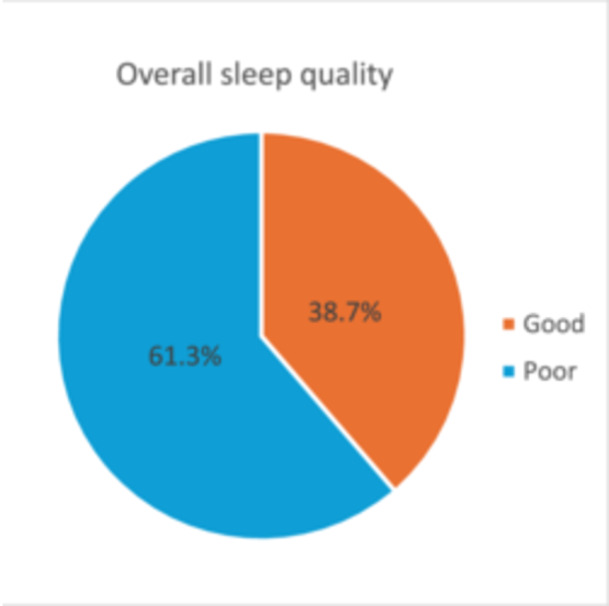
Overall sleep quality (good vs. poor) among study participants.

**Figure 2:**
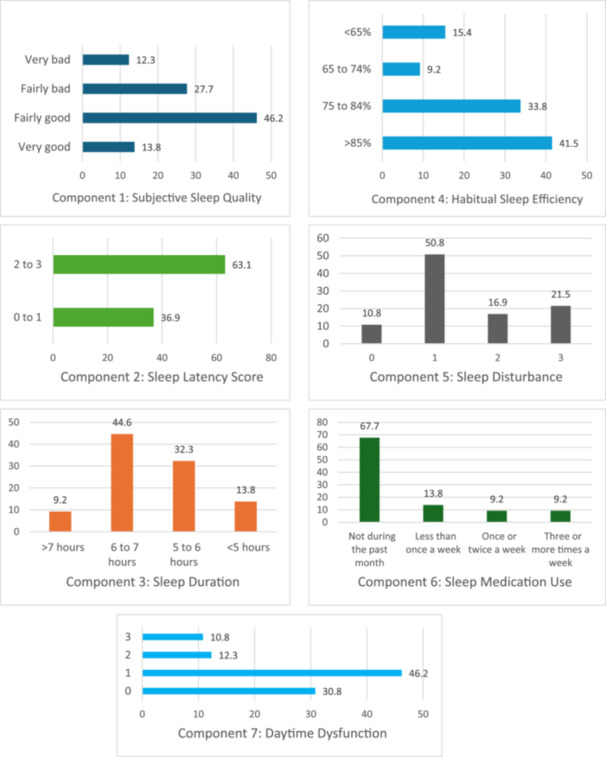
Summary of responses on the different component of Pittsburgh Sleep Quality Index.

Based on the PSQI, 61.3% (*n* = 217) of the study participants had poor sleep quality, and 38.7% (*n* = 137) of them had good sleep quality (Figure 1). Specifically, seven components of PSQI are shown in Figure 2. For example, approximately 40% of the participants reported their subjective sleep quality as either “fairly bad” (27.7%) or “very bad” (12.3%). Nearly half of the participants (46.1%) reported that they slept less than 7 h per night. Moreover, two‐thirds of the participants (67.7%) have not taken sleep medication during the last month, and 18.4% of them used sleep medication once or more than once a week (see Figure 2).

### 3.3 Sociodemographic, Behavioral, and Mental Health‐Related Correlates of Sleep Quality

Bivariate analysis shows that gender (*p* = 0.026), frequency of on‐call duty (*p* = 0.014), current smoking habits (*p* = 0.004), and depressive symptoms (*p* = 0.004) were significantly associated with sleep quality in our study sample (Table 2).

**Table 2:** Distribution of sleep quality (good vs. poor) by participants' demographic, occupation, behavioral, and mental health‐related characteristics (*n* = 354).

Variables	Sleep quality				*p* value
	Good		Poor		
	*n*	%	*n*	%	
Sex					**< 0.001**
Male	43	25.3	127	74.7	
Female	94	51.1	90	48.9	
Age (in years)					0.432
24–29	71	40.3	105	59.7	
30–40	42	40.0	63	60.0	
41–50	19	30/2	44	69.8	
> 50	5	50.0	5	50.0	
Marital status					0.264
Single	72	42.1	99	57.9	
Married	60	36.8	103	63.2	
Widow, divorced, or separated	5	25.0	15	75.0	
Having children					0.792
Yes	53	37.9	87	62.1	
No	84	39.3	130	60.7	
Income (monthly)					0.390
< 15,000 SAR	14	41.2	20	58.8	
15,001–20,000 SAR	74	35.7	133	64.3	
> 20,000 SAR	49	43.4	64	56.6	
Work classification					0.596
Resident	68	38.2	110	61.8	
Specialist	38	36.2	67	63.8	
Consultant	31	43.7	40	56.3	
Institution of work					0.803
Government	109	38.4	175	61.6	
Private	28	40.0	42	60.0	
Frequency of on‐call in the last month					**0.014**
One per week	70	42.7	94	57.3	
Two per week	31	47.7	34	52.3	
Three or four per week	36	28.8	89	71.2	
Current smoking status					**0.004**
Yes	8	18.6	35	81.4	
No	129	41.5	182	58.5	
Self‐reported body mass index					0.106
Underweight	17	30.9	38	69.1	
Normal weight	60	35.9	107	64.1	
Overweight/obese	60	45.5	72	54.5	
Having depressive symptoms					**0.004**
Yes	48	30.4	110	69.6	
No	89	45.4	107	54.6	
Having anxiety symptoms					0.314
Yes	47	35.3	86	64.7	
No	90	40.7	131	59.3	

*Note:* Bolded values represent statistically significant (*p* < 0.05).

**Figure 3:**
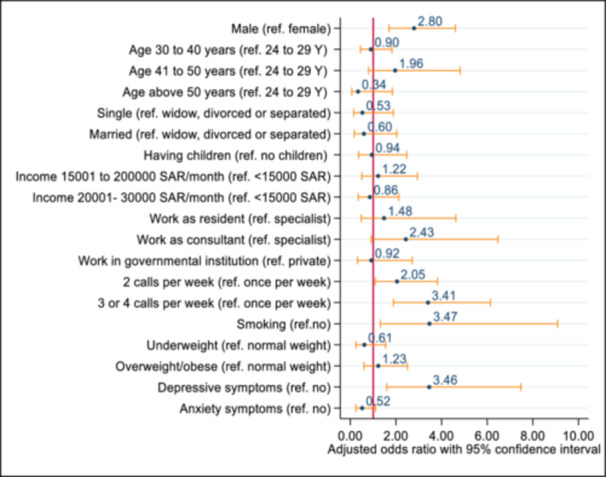
Odds ratio plot represents the sociodemographic, behavioral, and mental health‐related correlates of poor sleep quality. Each row's circular marker (blue color) and horizontal capped spike (yellow color) range represent the odds ratio and 95% confidence interval for a particular variable. Statistical significance was determined when the 95% confidence interval did not go beyond the reference margin (red color, odds ratio = 1).

In Figure 3, we demonstrated the sociodemographic, behavioral, and mental health‐related correlates of poor sleep quality by an odds ratio plot. The adjusted regression model was statistically suitable to estimate the predictors (*χ* ^2^ value = 8.60 and *p* = 0.3776).

Multivariate binary logistic regression analysis revealed that male participants had a 2.8 times higher likelihood of developing poor sleep quality compared to their female counterparts (adjusted odds ratio [AOR] = 2.80, 95% CI = 1.70–4.61, *p* < 0.001). Participants who had on‐call duties ≥ 2 times per week were at higher risk of developing poor sleep quality compared to those who had on‐call duties once per week (for two per week: AOR = 2.05, 95% CI = 1.09–3.82, *p* = 0.025; for three or four per week: AOR = 3.41, 95% CI = 1.89–6.14, *p* < 0.001). Smoker participants were more likely to have poor sleep than nonsmoker participants (AOR = 3.47, 95% CI = 1.32–9.08, *p* = 0.011). Participants with depressive symptoms had higher odds of developing poor sleep quality compared to their counterparts (AOR = 3.46, 95% CI = 1.60–7.48, *p* = 0.002) (see Table 3).

**Table 3:** Multivariate logistic regression model demonstrating the predictors of poor sleep quality among study participants.

Variables	Adjusted binary regression model		
	Odds ratio	95% Confidence interval	*p* value
Sex			
Male	2.8	1.70, 4.61	**< 0.001**
Female	Reference		
Age (in years)			
24–29	Reference		
30–40	0.90	0.45, 1.82	0.773
41–50	1.96	0.80, 4.82	0.141
> 50	0.34	0.06, 1.85	0.211
Marital status			
Single	0.53	0.15, 1.88	0.325
Married	0.60	0.17, 2.04	0.409
Widow, divorced, or separated	Reference		
Having children			
Yes	0.94	0.36, 2.47	0.902
No	Reference		
Income (monthly)			
< 15,000 SAR	Reference		
15,001–20,000 SAR	1.22	0.50, 2.94	0.664
> 20,000 SAR	0.86	0.35, 2.13	0.740
Work classification			
Resident	1.48	0.47, 4.63	0.502
Specialist	Reference		
Consultant	2.43	0.92, 6.47	0.075
Institution of work			
Government	0.92	0.311, 2.71	0.878
Private	Reference		
Frequency of on‐call in the last month			
One per week	Reference		
Two per week	2.05	1.09, 3.82	**0.025**
Three or four per week	3.41	1.89, 6.14	**0.000**
Current smoking status			
Yes	3.47	1.32, 9.08	**0.011**
No	Reference		
Self‐reported body mass index			
Underweight	0.61	0.24, 1.55	0.302
Normal weight	Reference	0.60, 2.50	0.577
Overweight/obese	1.23		
Having depressive symptoms			
Yes	3.46	1.60, 7.48	**0.002**
No	Reference		
Having anxiety symptoms			
Yes	0.52	0.24, 1.11	0.091
No	Reference		

*Note:* Bolded values represent statistically significant (*p* < 0.05).

### 3.4 Sleep‐Related Habits and Its Association With Poor Sleep Quality

**Figure 4:**
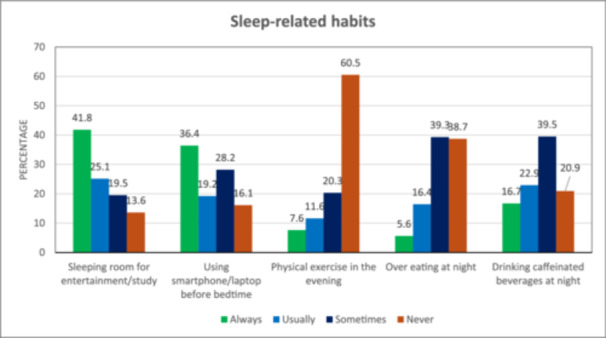
Sleep‐related habits of study participants.

Detailed information on participants' sleep‐related habits is depicted in Figure 4. A higher proportion of the participants reported they always use a sleeping room for entertainment and study (*n* = 148, 41.8%). More than one‐third of the participants reported that they always use a smartphone or laptop before going to bed (*n* = 129, 36.4%). Nearly two‐thirds of the participants stated that they never exercised in the evening (*n* = 214, 60.5%). Approximately 40% of the participants mentioned that they sometimes consume caffeinated beverages at night (*n* = 140, 39.5%).

In Table 4, adjusted binary regression analysis shows the association between sleep‐related habits and poor sleep quality among study participants. This adjusted regression model was statistically fitted (Hosmer and Lemenshow test: *χ* ^2^ value = 9.54 and *p* = 0.2987). This study revealed that participants who always used their smartphone or laptop before going to bed were more likely to have poor sleep quality than those who never used such (AOR = 3.15, 95% CI = 1.31, 7.60, *p* = 0.010).

**Table 4:** Binary regression analysis showing the association between sleep‐related habits and poor sleep quality among study participants.

Variables	Adjusted regression model		
	Odds ratio	95% Confidence interval	*p* value
Using the sleeping room for entertainment/study			
Always	1.45	0.44, 4.72	0.539
Usually	1.75	0.43, 7.14	0.437
Sometimes	1.28	0.37, 4.42	0.696
Never	Reference		
Using smartphone/laptop before bedtime			
Always	3.15	1.31, 7.60	**0.010**
Usually	2.07	0.80, 5.38	0.135
Sometimes	1.84	0.69, 4.89	0.222
Never	Reference		
Physical exercise in the evening			
Always	1.45	0.27, 7.70	0.659
Usually	1.95	0.69, 5.53	0.206
Sometimes	1.22	0.54, 2.77	0.636
Never	Reference		
Overeating at night			
Always	1.37	0.20, 9.31	0.748
Usually	1.15	0.48, 2.76	0.750
Sometimes	1.10	0.57, 2.12	0.772
Never	Reference		
Drinking caffeinated beverages at night			
Always	0.52	0.16, 1.67	0.273
Usually	0.54	0.15, 1.97	0.351
Sometimes	0.45	0.15, 1.32	0.144
Never	Reference		

*Note:* Bolded values represent statistically significant (*p* < 0.05).

## 4 Discussion

Sleep is a biological necessity required to maintain optimum health [2, 3], and it is one of the key features in determining a person's quality of life [32, 33]. However, poor sleep quality or sleep disturbances can cause major disruptions in human functionality. This consequence is also true for healthcare practitioners such as psychiatry physicians. Thus, using a cross‐sectional research approach, we investigated the status and determinants of poor‐quality sleep among Saudi psychiatry physicians.

The current study reported that nearly two‐thirds of the study participants (61.3%) were poor sleepers as per the PSQI criterion. This finding was similar to a previous study conducted among psychiatry residents in Brazil, reporting that 59.3% of participants had poor sleep quality according to the PSQI screener [34]. Our study found a higher percentage of poor sleep quality than a previous study carried out among 70 psychiatry residents in Italy (i.e., poor sleepers 44.3% based on PSQI criterion) [35]. The higher rate of poor sleep quality among psychiatry residents in Saudi Arabia is also confirmed by a Saudi‐based study investigated by AlSaif [13]. In his study, he reported that 25 of 31 psychiatry residents had poor sleep quality based on the PSQI, which means 80.6% were poor sleepers [13]. Our study indicates a high rate of poor sleep quality among psychiatry physicians, which calls for immediate intervention to improve their sleep quality. In particular, future qualitative investigations are especially recommended to uncover the causes of poor sleep quality among Saudi psychiatry professionals.

Due to the lack of similar research among physicians, the components of the PSQI can be compared to different subpopulations across the world. For example, in our study, 40% of the participants reported their subjective sleep quality as bad (bad = fairly bad + very bad), which is similar to a previous study conducted among college students in Hong Kong (39.7%) [36]. Furthermore, our study found that almost half of the participants slept < 7 h sleep per night. An Italian study reported that the average sleep duration per night among psychiatry residents was 6 h [35]. As sleep deprivation is associated with poor work performance, stress, and fatigue, residency programs sensitize psychiatry residents about the negative effects of sleep deprivation so that they can maintain adequate sleep. Another important finding of this study is that approximately 18.4% of the respondents took sleep medication for a minimum of once per week. These rates are lower than the Brazilian study, which reported that 54.2% of psychiatry residents used sleep medication [34]. It is worth mentioning that the abuse of sleeping pills is harmful to physical, mental, and cognitive well‐being. It can cause drowsiness, mental retardation, and driving performance, and so forth [37].

According to the regression estimate, male psychiatry physicians were more prone to develop poor sleep quality than their female residents. This may be justified by men's unhealthy lifestyles, such as poor diet, smoking, sedentary behavior, and social media addiction that make them more vulnerable to experience poor sleep [38, 39]. Gender differences in sleep quality can be compared by the evidence from other population groups. Similar to our findings, previous studies from Bangladesh [40] and Taiwan [41] reported that male students had a greater risk of developing poor sleep quality than female students. In contrast to our finding, female gender was connected with an increased likelihood of poor sleep quality among junior physicians in Pakistan [42] and physicians and nurses in a tertiary health care center in Saudi Arabia [22]. This could be explained by the notion that female physicians are more vulnerable to social and psychiatric difficulties, as well as decreased liveliness compared to general individuals, which could affect their sleep quality [30, 43]. Furthermore, the gender ratio of study samples may produce sex‐based risk differences in sleep quality. When comparing our findings with other investigations (female participants: 52.0%), the majority of study participants in Alghamdi et al.'s [22] study were female (70.4%) which may have influenced the study results. However, several studies could not draw any significant conclusions between the two genders in regard to their sleep quality [11, 13, 44]. Further qualitative or follow‐up studies are recommended to better understand gender differences in sleep quality among physicians in Saudi Arabia.

This study revealed higher frequency of on‐call duty (≥ 2 times per week) was associated with poor sleep quality among psychiatry physicians. This finding is comparable with previous studies [22, 45, 46]. Karhula et al. [45] showed that frequent on‐call duties (> 12 shifts/3 months) were associated with physicians' inadequate sleep. A recent systematic review reported that on‐call work at home was associated with lower sleep duration and poor sleep quality [46]. The on‐call duty may have a negative impact on physicians' family time, social life, and stress levels, further interrupting their sleep. So, it is strongly suggested to lower the frequency of on‐calls, shift work, and working hours so that physicians can maintain job‐related stress and get enough sleep.

In our study sample, psychiatry physicians who were current smokers had a higher risk of poor sleep than nonsmokers. This finding is similar to a previous study in Saudi Arabia, reporting current smoking habits were correlated with poor sleep quality among healthcare professionals [22]. This observed relationship is also aligned with various literature [22, 47, 48]. Multiple factors are responsible for hampering the sleep quality of smokers, such as chemical agents of tobacco products and physiological mechanisms. The physiological urge for nicotine during sleep may induce smokers to awaken, resulting in insomnia [49]. Furthermore, nicotine disrupts sleep latency and circadian rhythm, raising the likelihood of snoring and obstructive sleep apnea [50, 51]. Smoking cessation is vital for everyone, yet asking about smoking and suggesting to quit was uncommon among smoking physicians [52]. Policymakers should create evidence‐based smoking cessation programs for physicians and other healthcare professionals, which will enhance their health outcomes, including sleep quality, and allow them to participate in realistic tobacco control and preventive initiatives for the general public.

Another important finding of our study was that depressive symptoms were associated with poor sleep quality among study participants, which is consistent with previous studies from different countries and population groups [53, 54, 55]. Abdellah et al. [54] conducted a study among physicians in Saudi Arabia and showed that depression was significantly associated with poor sleep quality amid the COVID‐19 pandemic. One possible justification is that circadian preferences may play a significant influence on the relationship between sleep and depression [56]. Future longitudinal studies are recommended to understand the directionality of these parameters, whether depression causes sleep disturbances or sleep deprivation leads to depression. Additionally, our findings emphasize the necessity of developing measures for preventing major depression in Saudi psychiatric practitioners. It is evident that psychological issues (burnout, depression, anxiety, etc.) are prevalent among mental health professionals in Saudi Arabia [57]. Future research should focus on understanding the factors associated with mental health issues such as depression, anxiety, and stress among psychiatry physicians or psychiatrists, which will help policymakers develop targeted interventions to reduce their mental health burden and improve the quality of care for mental health patients.

In addition, we found participants who always used their smartphone or laptop before going to bed were at greater risk of poor sleep quality. This association is well‐documented and established in the literature [58, 59, 60, 61, 62, 63]. Electronic gadgets produce short‐wavelength blue light (380–495 nm), which hinders the formation of melatonin, a hormone that maintains circadian rhythms. As a result, using a smartphone or laptop before bedtime suppresses melatonin production and causes sleep disruptions [64, 65, 66]. Furthermore, using a mobile screen/laptop might create physical and psychological hyperexcitability, which contributes to a high arousal level and poor sleep quality [60, 67]. It is, therefore, imperative that governments and other organizations should sensitize people about the adverse effects of excessive and inappropriate use of technology‐based electronic devices so that they can maintain a healthy lifestyle, including adequate sleep.

Our study had several limitations. Due to the cross‐sectional design causal association cannot be established. We did not include any data on working experience and psychotropic consumption; future studies should consider these variables to address gaps. Further research should be planned to assess the effect of psychotropic intake on various sleep characteristics, such as insomnia and sleep apnea among physicians in Saudi Arabia. Generalization of the findings can be limited due to the nonprobability sampling approach. Because the study participants are psychiatric professionals who are generally well‐oriented with PHQ‐9 and GAD‐7, there may be some response biases; thus, the findings should be judged with caution. Due to the self‐reported nature of the outcome assessment, reporting biases may arise. Moreover, social desirability biases may be present in our sample.

## 5 Conclusion

In summary, poor‐quality sleep is extremely prominent among psychiatry physicians in Saudi Arabia. Male sex, higher on‐call duty, smoking habits, depression and smartphone/laptop use before bedtime were significantly associated with poor sleep quality in our study sample. These findings emphasize the need for sleep‐health promotion interventions for psychiatry physicians, as well as raising Saudis' awareness about poor sleep quality. Future longitudinal studies that incorporate additional cultural and lifestyle parameters are recommended to establish the causal factors of poor sleep quality among psychiatry physicians in Saudi Arabia.

## Author Contributions

**Najim Z. Alshahrani:** conceptualization, investigation, funding acquisition, writing–original draft, methodology, validation, visualization, writing–review and editing, software, formal analysis, data curation, resources, supervision, project administration. **Abdullah M. Alarifi:** funding acquisition, writing–original draft, methodology, visualization, validation, resources, formal analysis, project administration, data curation. **Wejdan Saqer Alotaibi:** investigation, funding acquisition, writing–original draft, methodology, software, formal analysis, data curation, resources. **Afnan Abdulrahman Alsayed:** data curation, resources, software, formal analysis, methodology, investigation, funding acquisition, writing–original draft. **Khalid Sultan Latif Alwasm:** data curation, supervision, software, formal analysis, writing–review and editing, methodology, funding acquisition, investigation. **Alaa Abdulkarim Alhunti:** data curation, supervision, software, formal analysis, methodology, writing–review and editing, funding acquisition, investigation. **Lana Alaa AlDahleh:** data curation, supervision, software, formal analysis, methodology, writing–review and editing, funding acquisition, investigation. **Meaad Mohammed A Alshahrani:** data curation, supervision, software, formal analysis, methodology, writing–review and editing, funding acquisition, investigation. **Abdalrhman M. Albeshry:** methodology, validation, writing–review and editing, investigation, funding acquisition, software, formal analysis, data curation, supervision. **Mohammed A. Aljunaid:** data curation, supervision, software, formal analysis, methodology, validation, writing–review and editing, funding acquisition, investigation.

## Ethics Statement

All study procedures involving human participants followed the Declaration of Helsinki. In addition, ethical authorization was taken from the Research Ethics Committee of Security Forces Hospital Program in the holy Capital (approval number: ECM#0546‐131222). Informed consent (i.e., electronic signature) was taken from all participants.

## Conflicts of Interest

The authors declare no conflicts of interest.

## Data Availability

The data supporting the findings of this investigation are accessible upon reasonable request from the corresponding author. All authors have read and approved the final version of the manuscript. Najim Z. Alshahrani had full access to all of the data in this study and takes complete responsibility for the integrity of the data and the accuracy of the data analysis.
